# Prediction of genome-wide effects of single nucleotide variants on transcription factor binding

**DOI:** 10.1038/s41598-020-74793-4

**Published:** 2020-10-19

**Authors:** Sebastian Carrasco Pro, Katia Bulekova, Brian Gregor, Adam Labadorf, Juan Ignacio Fuxman Bass

**Affiliations:** 1 Bioinformatics Program, Boston University, Boston, MA 02215 USA; 2 Research Computing Services, Boston University, Boston, MA 02215 USA; 3 Department of Neurology, Boston University School of Medicine, Boston, MA 02118 USA; 4 Biology Department, Boston University, Boston, MA 02215 USA

**Keywords:** Cancer, Computational biology and bioinformatics, Genetics

## Abstract

Single nucleotide variants (SNVs) located in transcriptional regulatory regions can result in gene expression changes that lead to adaptive or detrimental phenotypic outcomes. Here, we predict gain or loss of binding sites for 741 transcription factors (TFs) across the human genome. We calculated ‘gainability’ and ‘disruptability’ scores for each TF that represent the likelihood of binding sites being created or disrupted, respectively. We found that functional cis-eQTL SNVs are more likely to alter TF binding sites than rare SNVs in the human population. In addition, we show that cancer somatic mutations have different effects on TF binding sites from different TF families on a cancer-type basis. Finally, we discuss the relationship between these results and cancer mutational signatures. Altogether, we provide a blueprint to study the impact of SNVs derived from genetic variation or disease association on TF binding to gene regulatory regions.

## Introduction

Changes in gene expression caused by single nucleotide variants (SNVs) residing in transcriptional regulatory regions have been shown to cause phenotypic changes which may be adaptive or lead to disease^1–3^. The mechanisms of action of these SNVs include alterations in the binding of transcription factors (TFs), in the recruitment of RNA Polymerase II, in nucleosome positioning, and in DNA modifications. Among these, the creation and disruption of TF binding sites (TFBSs) is likely the main mechanism by which SNVs affect gene expression^2^.

Experimental methods to determine changes in TFBS affinities driven by SNVs include electrophoretic mobility shift assays (EMSA), chromatin immunoprecipitation followed by sequencing (ChIP-seq), and enhanced-yeast one-hybrid (eY1H) assays^4^. EMSA is a very low-throughput assay that tests one or few TFs and DNA sequences at a time, and requires TF purification or TF-specific antibodies. ChIP can be used to study differential TF recruitment by SNVs, but only tests one TF at time, is limited by the availability of high-quality TF-specific antibodies, and more importantly, requires cells to be heterozygous for the SNV of interest. eY1H assays, instead, can determine altered TF binding to a SNV by testing the full repertoire of TFs, but only test one SNV per experiment. Thus, current experimental methods are limited by the amount of SNVs and TFs they are able to test in a single experiment. Due to these limitations, prediction algorithms based on experimentally determined motifs have been developed for high-throughput prediction of altered TF binding by SNVs.

TFs binding preferences to DNA sequences, represented by position weight matrices (PWMs), have been used to predict the likelihood that a TF binds a DNA sequence of interest. These computational methods, that scan DNA regions to predict TFBSs, include FIMO^5^, RSAT^6^, Clover^7^, and ENCODE DREAM Challenge derived methods^8,9^, among others. In addition, methods have been developed to predict the impact of SNVs on TF binding, where scores of the mutated and wild-type DNA sequences are compared^10–15^. These methods have been used to predict the effect on TF binding of disease-associated SNVs such as those identified in genome-wide association and genetic studies^16–18^, and somatic mutations observed in tumor samples^19–22^. Furthermore, databases assessing the effect of known SNVs in the human population in gain/loss of TFBSs have been used to obtain insights into the effect of human variation on TF binding^13,23,24^. However, the effect of novel or unseen SNVs, such as rare variants and somatic mutations, on TF binding has not yet been determined. In this regard, a recent study evaluated the impact of tri-nucleotide cancer mutational signatures on TFBSs^20^. This study calculated the differential probabilities of gain and loss of TFBSs corresponding to each TF for each mutational signature based on calculating the effect of SNVs across DNA k-mers found in the human genome. However, this method precludes identifying the sets of TFBSs that are poised to be gained and lost by SNVs as it assumes a uniform distribution of k-mers across the human genome.

Here, we predict altered TFBSs genome-wide by in silico mutating all possible SNVs in every position in the human genome and determining gain and loss of TFBSs for 1898 PWMs corresponding to 741 human TFs. Using this resource, we show that the probability to gain (gainability) or disrupt (disruptability) a TFBS in gene regulatory regions widely differs between different TFs and TF families. We also show that functional *cis* expression quantitative trait loci (cis-eQTL) SNVs are more likely to perturb TFBSs than rare SNVs in the human population. Interestingly, the difference in disruptability is driven both by a higher probability of SNVs residing within TFBSs and a lower probability of retaining existing TFBSs by cis-eQTL versus rare SNVs. Finally, we show that somatic mutations in different cancer-types have differential effects on TFBSs between TF families and discuss how these profiles are related to distinct cancer mechanisms. Altogether, we provide a blueprint to study the impact of SNVs associated with genetic variation and cancer on TF binding.

## Results

### Estimating the effects of SNVs in creating and disrupting predicted TFBSs

**Figure 1:**
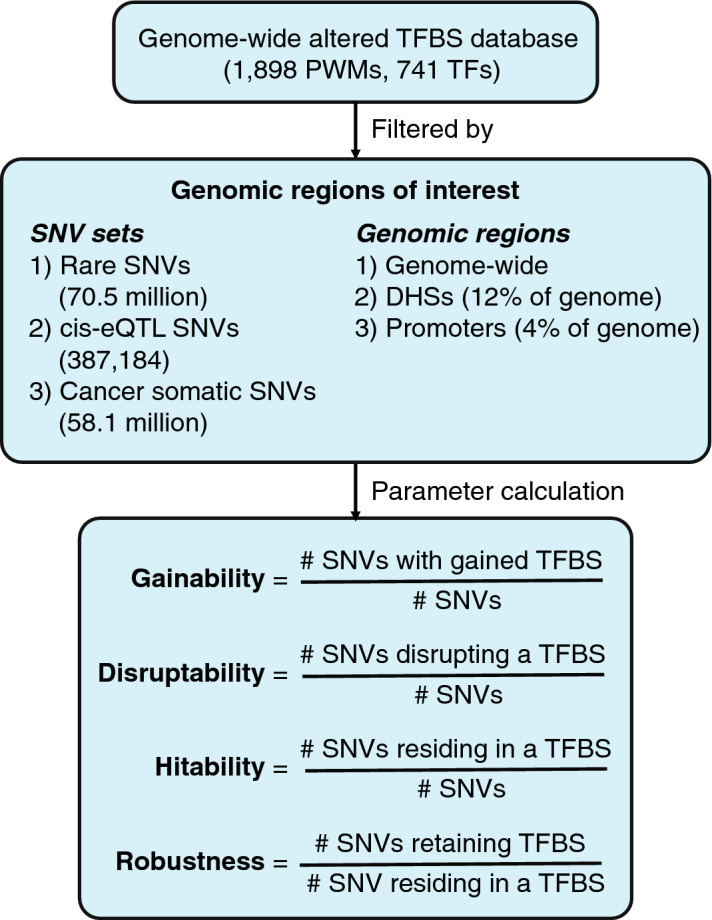
Outline of parameter calculation for different genomic regions. The effect of all possible SNVs in the human genome on TF binding was predicted based on 1898 PWMs available in CIS-BP. Genomic positions were then classified based on the type of genomic regions (e.g., promoter or DHS), sets of SNVs, or an intersection of both. Gainability, disruptability, hitability, and robustness were then calculated.

To predict the effect of each possible SNV in transcriptional regulatory regions on TF binding, we focused on DHS regions from the RoadMap Epigenomics Mapping Consortium^25^ (12% of the genome), which are generally associated with transcriptionally active or poised genomic regions. We calculated binding scores for 1898 PWMs available in CIS-BP^12^ corresponding to 741 human TFs, for each wild-type and alternative allele. We then predicted the effect of a SNV on a TF PWM by calculating its PWM score and compared this score to a pre-determined minimum PWM score threshold for predicted binding (see “Methods”). For each PWM-SNV combination, we determined whether the alternative allele created or disrupted a TFBS by calculating the ∆score (alternate – wild-type). A TF disruption is defined as a ∆score < 0, the wild-type allele score > score threshold, and the alternate allele score < score threshold. In contrast, A TF gain is defined as a ∆score > 0, the alternate allele score > score threshold, and the wild-type allele score < score threshold. Then, we defined two parameters for each TF and each type of genomic region: ‘gainability’ as the probability of a random SNV in the genomic region of study to create a binding site for a given TF, and ‘disruptability’ as the probability of a random SNV in the genomic region of study to disrupt an existing binding site for a given TF (Fig. 1). We also determined the gainability and disruptability scores genome-wide, and contrasted to that of DHS and gene promoter regions. We detected a wide range of distributions of gainability and disruptability scores for different TFs spanning five orders of magnitude which highly anti-correlated with the information content of the PWMs (Supplementary Figure S1). We found a strong correlation for both scores between the different genomic regions suggesting that there is no clear a priori preference for random SNVs to lead to gain or disruption of TFBSs both for regulatory regions and the whole genome (Supplementary Figure S2). Interestingly, we found a higher disruptability for AP-1 TFs (e.g*.*, FOS, FOSL1, FOSL2, JUN, JUNB, JUND), TAL1, and NFE2 in DHSs (which include both distal enhancer and gene promoters) than in promoter regions, consistent with previous findings that these TFs are enriched in binding to distal enhancer regions compared to proximal promoters^26,27^. Conversely, SP1-9 TFs display a higher disruptability in promoter regions, consistent with known roles of SP factors in regulating RNA Pol II recruitment to core promoters and regulating transcriptional activity.

**Figure 2:**
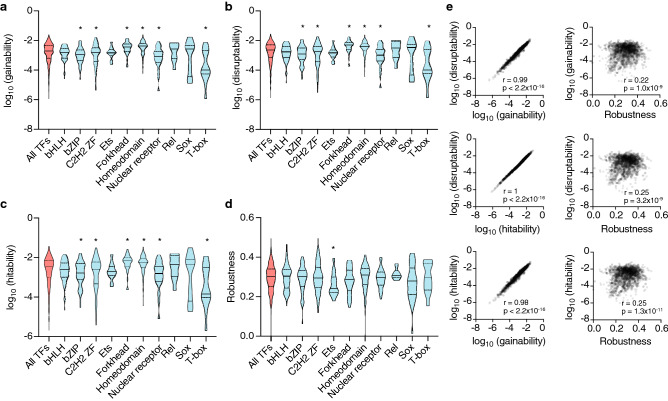
Prediction of the effect of SNVs on TF binding in DHSs. (**a**–**d**) The distribution of gainability (**a**), disruptability (**b**), hitability (**c**), and robustness (**d**) in DHSs were calculated for all TFs with available motifs in CIS-BP and binned by TF family. Significant differences for each parameter between a TF family and all TFs were calculated using a Mann–Whitney *U* test. *p < 0.05. (**e**) The correlation between each of the four parameters was estimated using the Pearson correlation coefficient.

TFs from the same DNA binding domain (DBD) family often have similar DNA binding preferences, in particular for certain families such as homeodomains, ETS factors, bHLH factors, and nuclear receptors, and are frequently different between TFs from different families^12^. Thus, we explored whether different TF families differ in gainability and disruptability scores. Indeed, we observed that homeodomain and forkhead TFs have a higher gainability than other TFs whereas bZIP, ZF-C2H2, nuclear receptors, and T-box have a lower gainability (Fig. 2a). A similar trend was observed for disruptability of these TF families (Fig. 2b), suggesting that homeodomains and forkhead TFs are more likely to be rewired by SNVs than other TF families. This is likely due to the short homeodomain and forkhead TF motifs, as we observed that gainability and disruptability are overall anti-correlated with PWM length and information content (Supplementary Figure S1).

The likelihood of SNVs disrupting TFBSs for a TF is influenced by two parameters: (1) hitability (i.e., the probability of a SNV residing within an existing TFBS), and (2) robustness (i.e., the chance that a SNV in a TFBS for such TF would not affect TF binding). In this way, disruptability is equal to the product of hitability and 1 – robustness. Of these two parameters, hitability has a larger impact on the difference in disruptability between TFs as it spans five orders of magnitude compared to robustness which spans only one order of magnitude (Fig. 2c,d). Interestingly, although hitability, gainability, and disruptability are all highly correlated with each other (Fig. 2e), in part driven by the information content of the PWMs (Supplementary Figure S1), robustness is lowly correlated with these parameters (Fig. 2e). Further, contrary to the other parameters, robustness is correlated to the information content per base in the PWM which has low variation between TFs, rather than the total information content (Supplementary Figure S1).

### Evidence of selection in noncoding rare SNVs

**Figure 3:**
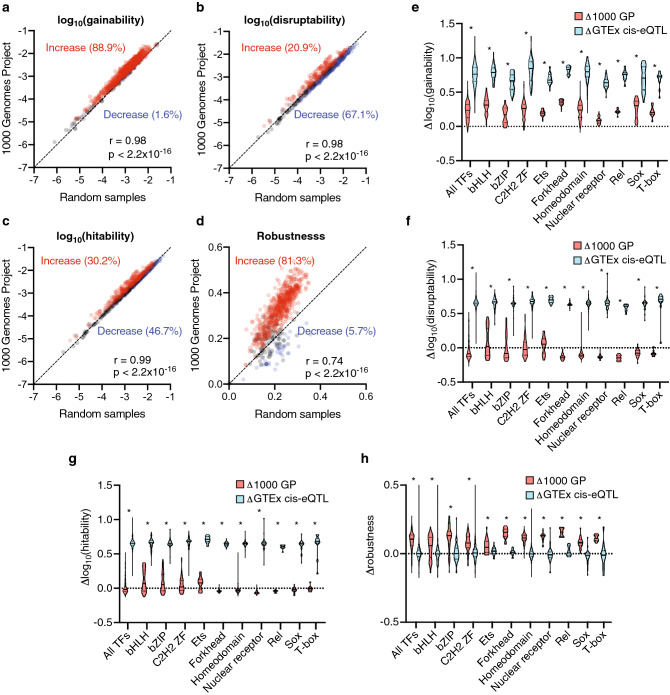
Differential parameter scores for rare and cis-eQTL SNVs. (**a**–**d**) Correlation between scores (robustness) or log_10_(scores) (gainability, disruptability, and hitability) derived from rare SNVs from the 1000 Genomes Project and the average of 100 random sets of 1,000,000 SNVs (Random samples). Correlation was determined by the Pearson correlation coefficient. Significantly enriched (red) and depleted (blue) TFs are highlighted. (**e**–**h**) Δscores or Δlog_10_(scores) (observed in set – reference) for each parameter for all TFs and specific TF families for rare and cis-eQTL SNVs. Significant differences between the rare and cis-eQTL scores were determined by a Mann–Whitney *U* test. *p < 0.05.

The human population displays high variability in genome sequence with more than 250 million SNVs being reported, most of which are rare in the population (minor allele frequency < 0.01)^28^. Most of these rare SNVs reside in noncoding regions of the genome potentially creating or disrupting TFBSs^1–3^. However, the vast majority of these rare SNVs are expected to be neutral and regulatory regions are likely depleted of SNVs under negative selection. Therefore, we hypothesized that rare SNVs present in the population would be depleted in those that alter TF binding, as changes in gene expression are expected to be evolutionarily constrained. To study the effect of rare genetic variation on TF binding, we analyzed rare SNVs from the 1000 Genomes Project^29^ located in DHS regions and determined gainability, disruptability, hitability, and robustness scores for each TF (Supplementary Table S2). We compared these parameters to the mean score for each PWM/TF from 100 random samplings of one million mutations based on the mutational frequency observed in the 1000 Genomes Project for each of the twelve possible SNV changes (4 nucleotides × 3 substitutions per nucleotide) (Supplementary Figure S3). Interestingly, 88.9% of the TFs show a significantly higher gainability score than the random samples (Fig. 3a). In contrast, 67.1% of the TFs show a significantly lower disruptability for the rare SNVs (Fig. 3b). These results suggest a selection of rare SNVs against disrupting existing TFBSs and a positive selection towards creating TFBSs.

We further calculated the hitability and robustness scores for rare SNVs to explore the mechanisms of the negative selection observed for disruptability. Strikingly, we found that even though hitability per TF is similar between rare SNVs and the random samples (Fig. 3c), rare SNVs show higher values for robustness for 81.3% of TFs (Fig. 3d). These results suggest that the negative selection towards TFBS disruption in rare SNVs is mainly driven by the selection for SNVs that, even though they may reside within existing TFBSs, they do not perturb TF binding.

### cis-eQTL SNVs display a high likelihood to create and disrupt TFBSs

Previous studies on cis-eQTLs have identified functional sets of SNVs in transcriptional regulatory regions associated with changes in target gene expression^30^. We compared the scores of cis-eQTL and rare SNVs for each parameter in this study (Supplementary Table S2) to the average of the random samples used to compare rare SNVs and generated Δscores (SNV group – random samples). We found high Δgainability and Δdisruptability scores for all TF families in the cis-eQTL SNV set compared to the Δscores for the rare SNV set (Fig. 3e,f). This suggests that cis-eQTLs are enriched in SNVs that create or disrupt TFBSs which likely contributes to their effect in differential gene expression. We further investigated the effects on cis-eQTLs disruptability and found that cis-eQTL SNVs lead to higher Δhitability and lower Δrobustness scores than rare SNVs (Fig. 3g,h). These findings suggest that the increased disruptability by cis-eQTLs SNVs is due to both an increase in SNVs being located in existing TFBSs and by affecting bases with higher information content within those TFBSs.

### Cancer somatic mutations display cancer- and TF family-specific effects on TFBSs

Cancer is characterized by the presence of somatic SNVs in tumors, more than 90% of which reside in noncoding regions of the genome^31^. It has been shown that different cancer-types display different mutational signatures driven by different mutation and DNA repair mechanisms^32,33^. Given the DNA binding specificity differences between TFs, we hypothesized that mutational signatures specific to different cancer-types may affect TFBSs differentially across TF families. To investigate this hypothesis, we selected SNVs located in DHS regions from 20 cancer-types from 2658 tumor samples from the Pan-Cancer Analysis of Whole Genomes (PCAWG) Consortium^34^ and calculated, for each TF, its Δgainability, Δdisruptability, Δhitability and Δrobustness scores relative to the background reference scores based on a uniform mutational frequency (all possible SNVs) in DHSs.

**Figure 4:**
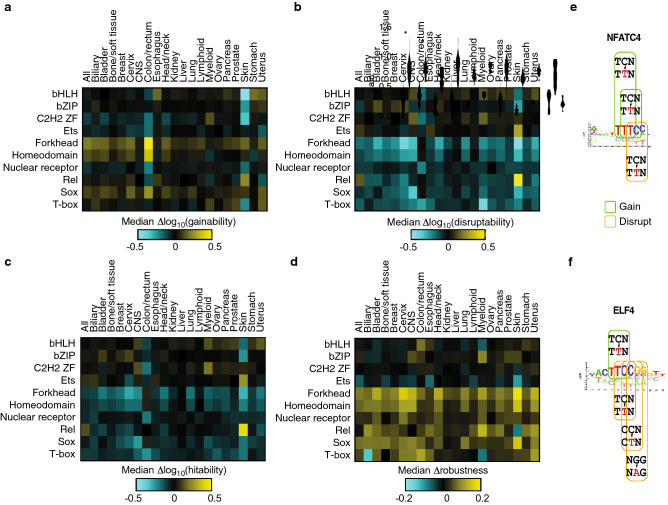
Effect of cancer somatic mutations on TFBSs. (**a**–**d**) Median Δscores or Δlog_10_(scores) for each TF family and cancer-type combination for gainability (**a**), disruptability (**b**), hitability (**c**), and robustness (**d**). (**e**,**f**) Motifs logos for NFATC4 (**e**) and ELF4 (**f**) and impact of melanoma mutational signatures on the gain and disruption of the corresponding motifs.

We found higher Δgainability scores for forkhead and Sox families across many cancer-types (Fig. 4a), with the highest enrichment in colon/rectum cancer. This is consistent with studies showing that the forkhead TFs FOXO3 and FOXA1, which have a 2 and 2.4-fold increase in gainability in colon/rectum cancer respectively, promote colon cancer proliferation^35^. Similarly, overexpression of FOXJ1 has been linked to progression of colorectal cancer by promoting translocation of β-catenin^36^. Sox TFs are also associated with cancer, including SOX11 that shows a 1.5-fold increase in gainability in breast cancer and that has been correlated with breast cancer growth and invasion^37^. Whether these results support a positive selection for gaining and maintaining forkhead and sox TFBSs in cancers or whether this is associated with specific cancer mutational signatures, remains to be determined.

Other associations for Δgainability scores between TF families and cancer-types are more specific. For example, we found gain of homeodomain TFBSs to be highly enriched in colon cancer (Fig. 4a). Indeed, HOXA3, a homeodomain TF that shows a 1.5-fold increase in gainability, has been shown to promote colon/rectum cancer^38^. Other TFs from the homeodomain subfamilies HOXB and HOXD have also been found to be up-regulated in cancer^39,40^, displaying an average 2.8 and 2.4-fold increase in gainability across the subfamily, respectively. Furthermore, skin cancer shows an enrichment in gain of rel TFBSs, which is mainly driven by the NFAT subfamily. In particular, NFATC3 (3.8-fold increase in gainability) is highly expressed in skin cancer and is associated with cell transformation and tumor growth in this cancer-type^41^. Conversely, we found a depletion to gain TFBSs from the bHLH, bZIP, and ZF-C2H2 families in skin cancer. In particular, we found that all of CREB TFs from the bZIP family show a negative Δgainability in skin cancer, where these TFs have been reported to inhibit tumor growth and metastasis^42^. In addition, ZBTB7A, a ZF-C2H2 TF with a 2.3-fold decrease in gainability in skin cancer, suppresses melanoma metastasis^43^.

In contrast to Δgainability, we found negative Δdisruptability scores for forkhead, homeodomain, nuclear receptor, rel, sox and T-box families across most of the 20 cancer-types analyzed (Fig. 4b). These results suggest a negative selection towards disrupting TFBSs for these families. Contrary to what we observed for rare SNVs where the reduced Δdisruptability was associated to an increase in Δrobustness, the reduced disruption for cancer mutations is associated with both an increase Δrobustness and a reduced Δhitability, suggesting negative selection (Fig. 4c,d). The main exceptions having a higher Δdisruptability score correspond to rel and ETS TFs in skin cancer, many of which have been associated with melanoma. This is consistent with the frequency of triplets matching the mutational signatures of melanomas (TCN → TTN and CCN → CTN)^33^ within motifs of rel factors such as NFATC4 (Fig. 4e) and ETS factors such as ELF4 (Fig. 4f). Altogether, our results suggest that cancer mutations lead to a net increase in TF binding sites for forkhead, homeodomain, nuclear receptor, rel, sox and T-box families.

**Figure 5:**
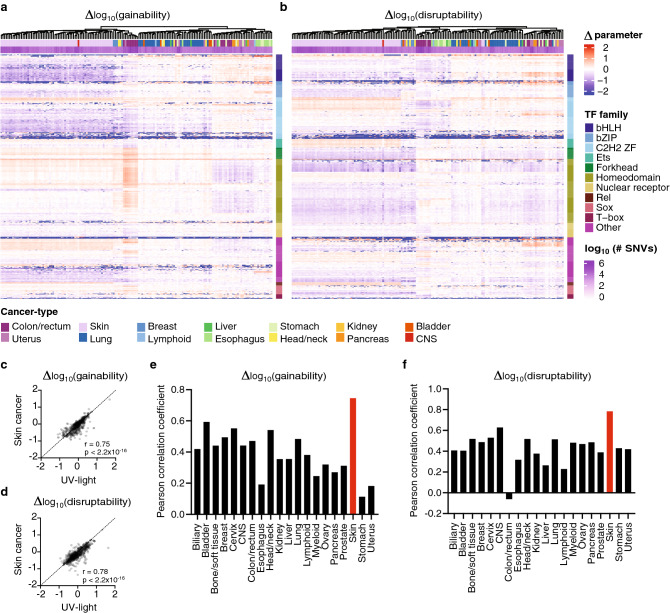
Effect of cancer somatic mutations in individual cancers on Δgainability and Δdisruptability. (**a**,**b**) For cancer samples with at least 5000 SNVs in DHS regions, we determined for each TF the Δlog_10_(gainability) (**a**) and Δ log_10_(disruptability) (**b**) scores. Samples were clustered using complete linkage clustering based on euclidian distance, and TFs were clustered by TF families. Cancer-types are indicated at the top and TF families are indicated at the right of each heatmap, respectively. (**c**,**d**) Correlation between UV-light-derived Δlog_10_(gainability) (**c**) and Δ log_10_(disruptability) (**d**) scores for each TF to those observed in skin cancer. Correlation calculated by the Pearson correlation coefficient. (**e**,**f**) Pearson correlation coefficients between UV-light-derived Δlog_10_(gainability) (**e**) and Δ log_10_(disruptability) (**f**) scores for each TF to those observed in each cancer-type.

Different tumors, even from the same cancer-type, can have different mutational signatures. Thus, we determined the Δgainability and Δdisruptability profile for 162 highly mutated tumors (> 5000 SNVs in DHSs) across 741 TFs. We observed a similar overall clustering pattern across tumors (Fig. 5a,b). Interestingly, all highly mutated skin cancer samples clustered together showing a similar pattern of gain and loss of TFBSs. This pattern is highly correlated to that of SNVs introduced by treating cell lines with UV light (Δgainability, r = 0.75, p < 2 × 10^–16^ and Δdisruptability, r = 0.78, p < 2 × 10^–16^) (Fig. 5c,d), consistent with UV light being a major mutational driver of skin cancer SNVs. This correlation with UV light induced mutations is higher for skin cancer mutations than for mutations identified in any other cancer-type evaluated (Fig. 5e,f). Surprisingly, colon/rectum tumor show two subtypes, where one subtype shows depletion of bZIP, bHLH and C2H2 zinc finger TFs and an enrichment of homeodomain TFs and the other subtype shows the opposite profile for both Δgainability and Δdisruptability (Fig. 5a,b). The origin of these colon/rectum tumor subtypes remains to be determined.

## Discussion

In this study, we predicted altered TFBSs obtained by in silico mutating all possible SNVs across the genome. Using this resource, we determined the gainability, disruptability, hitability, and robustness scores for 741 TFs across the genome, promoters, and DHS regions. We found differences in gainability and disruptability scores between TF families. For example, we found lower gainability and disruptability values for bZIP, C2H2 ZF, nuclear receptors, and T-box, showing that binding sites for these TF families are less likely to be affected by SNVs. In contrast, forkhead and homeodomain display higher scores for both gainability and disruptability, suggesting a higher rewiring potential of the gene regulatory networks controlled by these TFs. Whether in vivo binding site occupancy for these TFs is actually rewired across evolution or between individuals in the human population, remains to be determined. Given the broad distribution of scores even within TF families, a more granular classification of TFs such as that provided by TFClass^44^ may reveal further differences between TF family subclasses.

We showed that functional cis-eQTL SNVs are more likely to perturb TFBSs than rare SNVs in the human population. In addition, we observed that somatic mutations in cancer have differential effects on TFBSs for multiple TF families and discuss how these profiles are related to distinct cancer mechanisms. In addition, our results can be implemented further in methods to identify functional SNVs in sequencing data, as our estimated probabilities can be used as background probabilities to compare germline or somatic mutations associated with disease in a given cohort.

By comparing the genome-wide gainability and disruptability scores calculated for each TF to the respective scores for the corresponding TFs calculated based on promoter and DHS regions, we found that scores across TFs between different genomic regions are highly correlated. One explanation for this observation is that SNVs may affect TFBSs across the genome in a similar manner, independent of the genomic function. Another explanation, is that considering a coarse-grained comparison (e.g., all promoters and DHSs) would average differences that likely exist between subsets of regions. It is important to note that the parameters described in this manuscript are based on predicted TFBSs as the goal is to provide scores in the absence of epigenetic factors to provide a background for comparisons between sets of regions or SNVs. The scores determined in TF occupied sequences in each region, in particular regions of closed chromatin, may be different to the ones predicted across the whole genome.

By analyzing the parameter patterns of rare SNVs we showed that 88.9% of TFs showed increased gainability. However, this increase is significantly lower than the Δgainability values calculated for cis-eQTLs SNVs which correspond to expression perturbing SNVs. In contrast, 67.1% of TFs showed a decrease in disruptability by the rare SNVs, whereas the cis-eQTL SNVs displayed an increase in disruptability scores. Interestingly, this difference is driven by two factors: a higher likelihood of cis-eQTL SNVs to reside within a TFBS and a higher likelihood of rare SNVs that land in a TFBS to retain it. These results can be explained by most rare SNVs in the population being neutral, not affecting gene expression. Altogether, this suggests a higher selective pressure in rare SNVs to maintain existing TFBSs which function together with other TFs within specific cis regulatory logics, while gain of TFBSs can provide evolutionary plasticity. These results are consistent with previous findings involving rare SNVs that reside in gene regulatory regions in the human population being under selective pressure and depleted of SNVs with high information content bases within TF binding motifs^45^.

Analysis of somatic mutations from the PCAWG cohort revealed negative Δdisruptability scores for forkhead, homeodomain, nuclear receptor, rel, sox, and T-box across most of the cancer-types analyzed. These results are consistent with previous findings showing a selective pressure for maintaining existing TFBS in breast, liver and lung cancer-types^46^. This study also reported a negative selection towards creating TFBSs in these three cancer-types. Although, we did observe reduced gain for multiple combinations of TF families and cancer-types, we also observed several cases increased gain of TFBSs. For example, we found increased gain of sox and homeodomain TFBSs, which have previously been found to be associated with higher match motif scores by cancer-associated SNVs compared to the reference allele^47^. The differences observed between cancer-types and TF families likely arise from the different mutational signatures associated with the different types of cancers, as previously suggested^20^.

To our knowledge, this is the first study that predicts the effect of all possible SNVs on TF binding. The gainability, disruptability, hitability and robustness parameters calculated for each TF provide a powerful resource to predict the effect of SNVs on TF binding and provide a background for further studies in specific transcriptional control regions or produced by SNVs present in specific patient cohorts. Other applications of this resource include studying the potential of repetitive elements as latent reservoirs of TFBSs and uncovering the role of other disease associated SNV sets and carcinogen signatures. Ultimately, the integration of other datasets such as TF dimer motif specificities, TF motifs in the context of nucleosomal DNA^48^, and the inclusion of new TF motifs as they become available, will lead to a more comprehensive model of the effect of SNVs on TFBSs.

## Methods

### Generation of the altered TF binding site database

To predict the effect of all possible SNVs in the human genome on TF binding, for each possible SNV and each TF with available PWMs, we calculated the binding score for the reference and alternate SNV alleles. We downloaded 1898 PWMs corresponding to 741 human TFs from CIS-BP^12^ on April 3, 2018 and their respective TF family. Given a PWM of length *n* and a genomic position (hs37d5 from the 1000 Genome Project), for each of the *2n-1* DNA sequences on each strand of length n that overlap with the genomic position, we calculated a TF binding score using the function:

$$ F(s,\;M) = \sum\limits_{i = 1}^{n} {\log \left( {\frac{{M_{{s_{i} ,i}} }}{{b_{{s_{i} }} }}} \right)} $$

where *s* is a genomic sequence of length *n*, *M* is the PWM with *n* columns and each column in *M* contains the frequency of each nucleotide in each position *i* = 1,…,*n*, and $b_{{s_{i} }}$ is the background frequency of nucleotide *s*_*i*_ (assuming a uniform distribution). The highest score obtained for the 4*n − *2 sequences was assigned as the binding score corresponding to the PWM for the reference or alternate SNV alleles. Significant scores were selected and reported based on TFM-p-value^49^ score thresholds determined using a significance level of α = 10^–4^. This method was applied for each reference position and the three possible alternate SNVs for the complete genome (hs37d5) to create the altered TFBS database, a genome-wide catalogue of predicted SNV-PWM effects. A custom program was written in C and CUDA to generate the dataset (https://github.com/fuxmanlab/altered_TFBS). The program was executed on Nvidia GPUs that are available on the Boston University Shared Computing Cluster (SCC). The 6.1 Tb dataset was stored in a compressed Parquet format on a 320-core Hadoop cluster that is also part of the SCC. In addition, a query system was developed using Python and PySpark that was run on the BU Hadoop cluster. The query system was used to search either a set of SNVs from a variant calling format (VCF) file (e.g., rare SNVs or somatic mutations), or all possible SNVs from genomic regions in BED files (e.g., promoter or DHS regions). In both cases, the query reports the PWM scores for each reference/alternate genomic position pair where at least one of the alleles has a significant score for the given PWM. As an example, a query consisting of the human promoter coordinates from a BED file took about 60 min to complete on the Hadoop cluster.

### Genomic region definitions

The hs37d5 human genome, downloaded from the Sanger Institute (November 2, 2018), was used as reference. Promoters were defined as regions from − 2000 to + 250 bp from all transcription start sites (TSSs) from protein coding genes available at GENCODE 19 version (June 14, 2018)^50^ and correspond to 4% of the human genome. We used the R package IRanges^51^ and BEDTools^52^ to extract promoter coordinates and DNA sequences. We identified 2,319,494 DHS genomic coordinates (median length 97 bp) by taking the union of DHS regions from all samples of the Roadmap Epigenomics Mapping Consortium (July 31, 2019)^53^ which correspond to 12% of the human genome.

### Generation of reference parameters for altered TF binding in genomic regions

SNVs may affect TF binding by either creating or disrupting TFBSs. Therefore, we defined two parameters to estimate these effects for each given TF-PWM: gainability and disruptability. Gainability was defined as the ratio between the number of SNVs that lead to gain of TFBSs and the total number of SNVs that are not located within existing TFBS for the given PWM. This corresponds to the probability of creating a TFBS for a given PWM for the set of SNVs analyzed assuming equal likelihood of nucleotide changes. Disruptability was defined as the ratio between the number of SNVs that disrupt a TFBS and the total number of possible SNVs. This corresponds to the probability of a SNV disrupting an existing TFBS for a given PWM assuming equal likelihood of nucleotide changes. Disruptability can be divided into two components: hitability, which is the probability of a random SNV residing within a TFBS corresponding to the PWM; and robustness, which is the probability of a SNV that resides within a TFBS to retain the TFBS. Thus, disruptability corresponds to the hitability multiplied by 1 – robustness of a PWM. In the case of TFs with multiple PWMs, we used the median score across PWMs as the representative one for each parameter. The four parameters (gainability, disruptability, hitability, and robustness) were calculated for each TF for the human genome, promoters, and DHS regions (Supplementary Table S1). TFs were grouped by TF families according to the CIS-BP TF family classification and only families with ten or more TF members were selected for this study.

### Analysis of parameter scores for rare and cis-eQTL SNVs

Rare SNVs (minor allele frequency < 0.01) were downloaded from the 1000 Genomes Project^29^ in vcf format (October 1, 2019). BEDTools intersect function was used to select SNVs in promoters or DHS regions. Gainability, disruptability, hitability, and robustness scores were calculated as described above (Supplementary Table S2). For DHS regions, we calculated the correlation of each TF score derived from rare SNVs against an average of 100 random samples of 1,000,000 mutations matching the mutational frequency of each of the twelve types of SNV changes (4 nucleotides × 3 substitutions per nucleotide) in the 1000 Genomes Project set (see below and Supplementary Figure S3). In addition, we downloaded finely mapped cis-eQTL SNVs from GTEx^30^ (October 10 2020) reported by CaVEMaN^54^ and DAPG methods^55^. BEDTools intersect function and a custom R script were used to obtain unique cis-eQTL SNVs located in promoter and DHS regions that were identified by both cis-eQTL prediction algorithms. Then, gainability, disruptability, hitability, and robustness scores were calculated for the cis-eQTL SNVs (Supplementary Table S2). To determine whether the altered TF binding parameters were different than expected by chance between rare and cis-eQTL SNVs, we subtracted the individual scores for each TF to the reference set generated from a random sampling model (see below) to calculate Δscores for gainability, disruptability, hitability, and robustness.

### Estimation of TFBS parameters derived from a random SNV sample

Scores for gainability, disruptability, hitability, and robustness derived from a random sample of SNVs were generated to compare with scores determined for rare and cis-eQTL SNVs. One million random SNVs were selected in DHS regions matching the frequency of the twelve possible mutations from the rare SNVs in the 1000 Genomes Project. One hundred random samples were generated and the four parameters per sample were calculated for each PWM as previously discussed. For each parameter the average values for each PWM across the one hundred random samples was determined and used as reference to compare to scores determined based on rare and cis-eQTL SNVs.

### Calculation of parameters for cancer somatic and carcinogen SNVs

Somatic SNVs were obtained from 2658 whole genome sequenced samples from the PCAWG cohort across 20 cancer-types^21^. For each cancer-type, we combined the SNVs across its associated samples and generated a unique set of SNVs per cancer-type. BEDTools intersect function was used to extract SNVs in DHS regions for each cancer-type. The observed gainability, disruptability, hitability, and robustness scores were calculated for each TF (Supplementary Tables S3 and S4) and were subtracted by their corresponding score from the reference set of all possible SNVs in DHS regions. This resulted in Δscores for each PWM-cancer-type combination. We also calculated the median Δscore for each TF family and generated heatmaps in Prism version 8.3.1. Furthermore, we calculated the observed Δscores for gainability and disruptability for the 741 TFs for individual samples having more than 5000 SNVs located in DHSs. Clustered heatmaps comparing Δscores for individual samples and TFs were generated using complete linkage clustering based on euclidian distance using the R package ComplexHeatmap^56^. Finally, we downloaded SNVs caused by UV-light^57^ and these SNVs were filtered to obtain Δscores for each parameter in DHS regions as described for the PCAWG analysis. We calculated the correlation of the UV-light derived Δscores for gainability and disruptability to the corresponding Δscores from each cancer-type in PCAWG samples.

### Statistical analysis

Custom R scripts and Prism were used for statistical analysis. Correlation tests were performed using the Pearson correlation coefficient and group comparisons were performed using Kruskal–Wallis rank-sum test.

## Supplementary information

Supplementary Figure S1.

Supplementary Figure S2.

Supplementary Figure S3.

Supplementary Table S1.

Supplementary Table S2.

Supplementary Table S3.

Supplementary Table S4.

Supplementary Information.

## Data Availability

The data used in the analysis of this paper are provided as Supplementary tables. Scripts used in this manuscript are available in https://github.com/fuxmanlab/altered_TFBS
