# Publisher Correction: Prediction of genome-wide effects of single nucleotide variants on transcription factor binding

**DOI:** 10.1038/s41598-021-83094-3

**Published:** 2021-02-03

**Authors:** Sebastian Carrasco Pro, Katia Bulekova, Brian Gregor, Adam Labadorf, Juan Ignacio Fuxman Bass

**Affiliations:** 1 Bioinformatics Program, Boston University, Boston, MA 02215 USA; 2 Research Computing Services, Boston University, Boston, MA 02215 USA; 3 Department of Neurology, Boston University School of Medicine, Boston, MA 02118 USA; 4 Biology Department, Boston University, Boston, MA 02215 USA

Correction to: *Scientific Reports*, 10.1038/s41598-020-74793-4, published online 19 October 2020

This Article contains an error in Figure 4 where panel b is rendered incorrectly.

The correct Figure 4 appears below as Figure 1.

**Figure 1:**
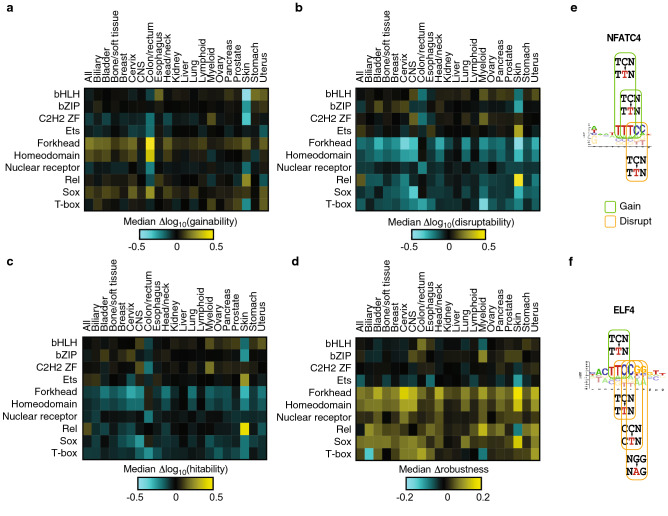
A correct version of the original Figure 4.

