# Supplementary figures and images for: Prediction of genome-wide effects of single nucleotide variants on transcription factor binding
Source: Sci Rep. 2020 Oct 19;10:17632. doi: 10.1038/s41598-020-74793-4 (PMC7572467; doi:10.1038/s41598-020-74793-4)

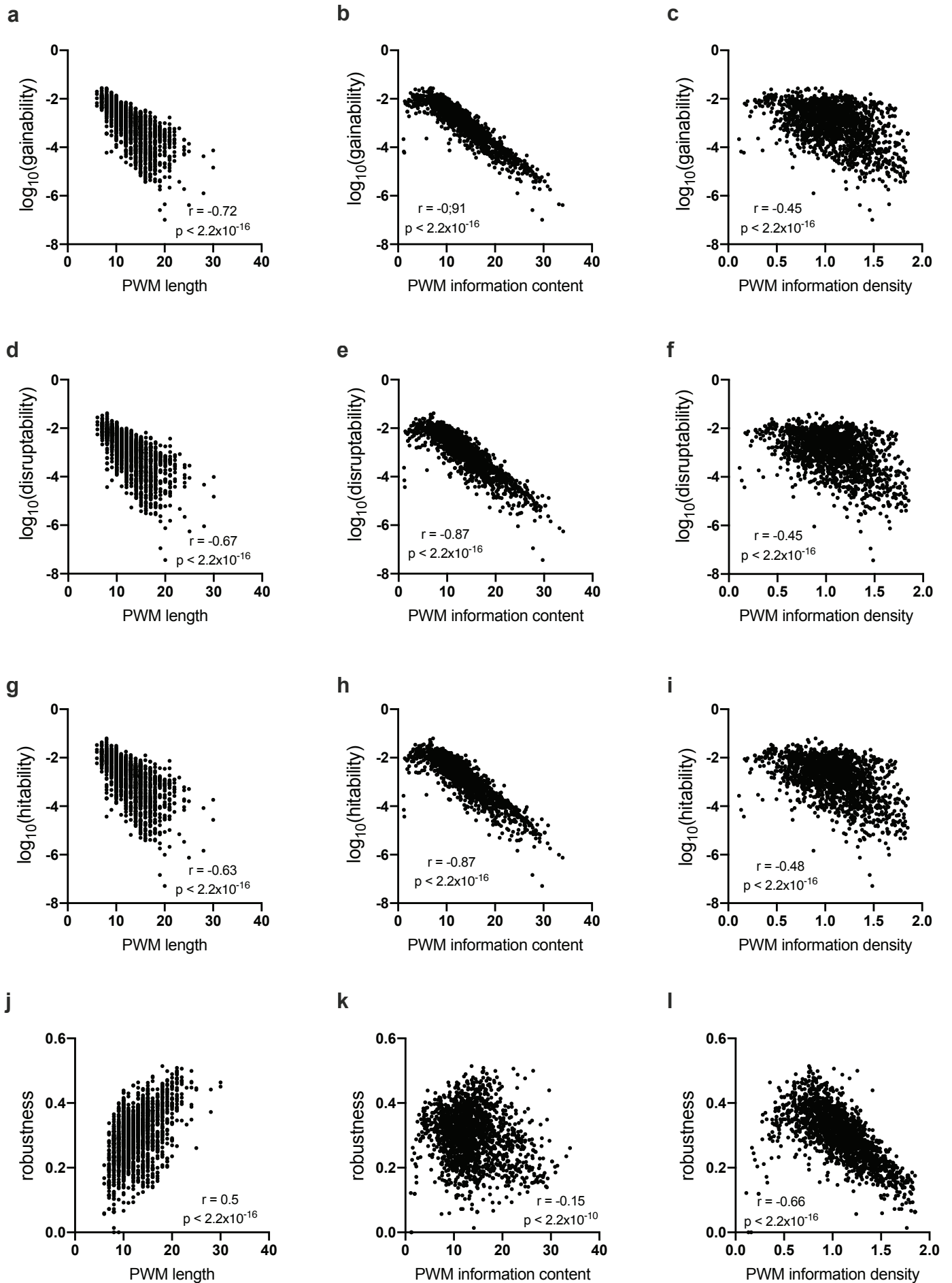

Supplement: Supplementary file 1 — Supplementary Figure S1. [file 41598_2020_74793_MOESM1_ESM.pdf]

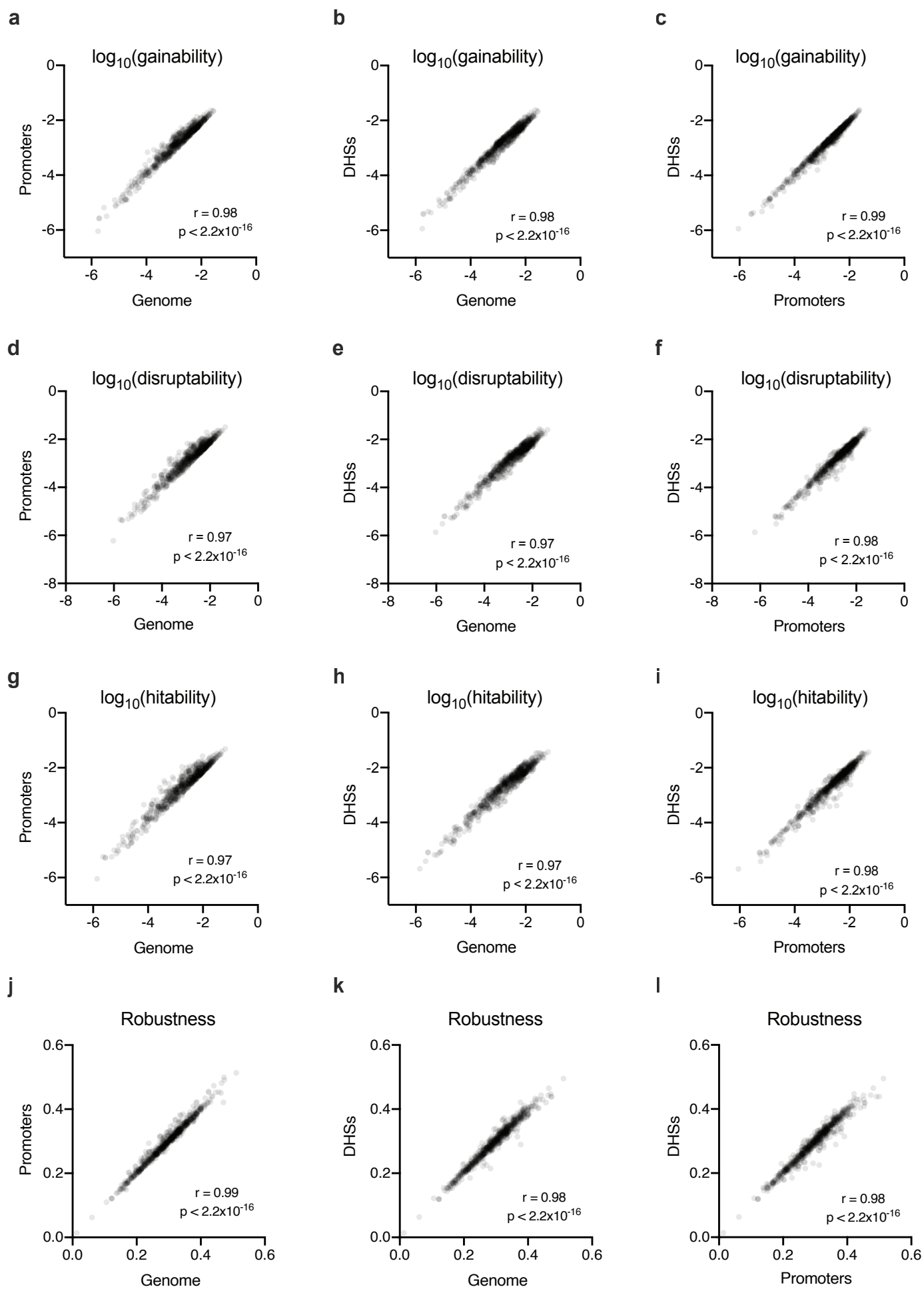

Supplement: Supplementary file 2 — Supplementary Figure S2. [file 41598_2020_74793_MOESM2_ESM.pdf]

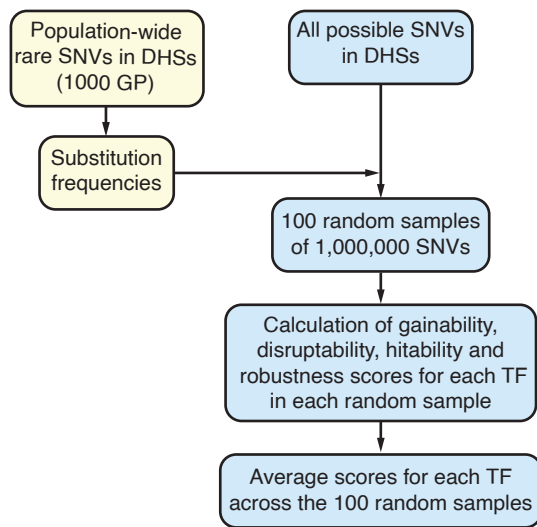

Supplement: Supplementary file 3 — Supplementary Figure S3. [file 41598_2020_74793_MOESM3_ESM.pdf]
