# Supplementary material for: Prediction of genome-wide effects of single nucleotide variants on transcription factor binding
Source: Sci Rep. 2020 Oct 19;10:17632. doi: 10.1038/s41598-020-74793-4 (PMC7572467; doi:10.1038/s41598-020-74793-4)
Supplement: Supplementary file 8 — Supplementary Information. [file 41598_2020_74793_MOESM8_ESM.docx]

**Prediction of genome-wide effects of single nucleotide variants on transcription factor binding**

Sebastian Carrasco Pro^1^, Katia Bulekova^2^, Brian Gregor^2^, Adam Labadorf^1,3^, Juan Ignacio Fuxman Bass^1,4*^

^1^Bioinformatics Program, Boston University, Boston MA 02215

^2^Research Computing Services, Boston University, Boston MA 02215

^3^Department of Neurology, Boston University School of Medicine, Boston MA 02118

^4^Biology Department, Boston University, Boston MA 02215

* Correspondence to: [fuxman@bu.edu](mailto:fuxman@bu.edu)

**Supplementary Figure Legends**

**Supplementary Figure S1**. Correlation between log_10_(scores) for gainability (**a-c**), disruptability (**d-f**), hitability (**g-i**) and socre for robustness (**j-l**) and PWM length, PWM information content, and PWM information density. Correlation determined by the Pearson correlation coefficient.

**Supplementary Figure S2.** Correlation between genomic regions (genome, promoters, DHS) for log_10_(scores) for gainability (**a-c**), disruptability (**d-f**), hitability (**g-i**) and robustness (**j-l**).

**Supplementary Figure S3.** Outline of calculation of 100 random samples to use as reference for rare and cis-eQTL SNVs.

**Supplementary Tables**

**Supplementary Table S1**. Reference parameter scores for each PWM and TF by genomic region.

**Supplementary Table S2**. Parameter scores for each PWM and TF determined based on rare and cis-eQTL SNVs.

**Supplementary Table S3**. Parameter scores for each PWM by cancer-type and TF.

**Supplementary Table S4**. Altered TF binding effect quantification per cancer-type sample used to calculate cancer associated parameters.
